# Does body mass index independently affect functional outcomes after conservatively treated distal radius fractures? a retrospective cohort study

**DOI:** 10.1186/s12891-026-09879-7

**Published:** 2026-04-22

**Authors:** Emin Can Balcı, Anıl Şüküroğlu

**Affiliations:** Department of Orthopedics and Traumatology, Bağcılar Training and Research Hospital, University of Health Sciences, Istanbul, Türkiye

**Keywords:** Distal radius fracture, Conservative treatment, Body mass index, Obesity, DASH score, Functional outcome

## Abstract

**Background:**

Obesity has been associated with inferior outcomes in several musculoskeletal conditions; however, its independent effect on functional recovery after conservatively treated distal radius fractures remains uncertain. This study aimed to evaluate whether body mass index (BMI) independently influences long-term functional outcomes following nonoperative management of distal radius fractures.

**Methods:**

This retrospective cohort study was conducted at a single tertiary trauma center between January 2023 and December 2025. During the study period, 188 adult patients were treated for distal radius fractures, of whom 141 were managed conservatively. Among these, 129 patients with complete 12-month Disabilities of the Arm, Shoulder and Hand (DASH) scores were included in the final analysis. Fractures were classified according to the AO system. BMI was analyzed as both a continuous variable and according to World Health Organization categories. Nonparametric tests and Spearman correlation were used for univariate analyses. Multivariable linear regression was performed to evaluate the independent association between BMI and 12-month DASH scores after adjustment for age, sex, and fracture type.

**Results:**

The mean age was 50.3 ± 15.3 years, and the mean BMI was 27.6 ± 4.5 kg/m². The median 12-month DASH score was 11.25 (interquartile range [IQR], 6.75–22.50). BMI demonstrated a weak positive correlation with 12-month DASH scores (ρ = 0.255, p = 0.0036), and obese patients exhibited higher DASH scores compared with normal-weight patients (p = 0.007). However, in multivariable analysis, BMI was not independently associated with 12-month DASH scores (β = 0.283, p = 0.201). Increasing age (β = 0.307, p < 0.001) and AO type B/C fractures (β = 6.777, p = 0.008) were independently associated with worse functional outcomes.

**Conclusion:**

Higher BMI was associated with worse unadjusted functional outcomes but did not independently predict 12-month DASH scores after adjustment for age and fracture severity. These findings suggest that BMI alone may not be a reliable prognostic indicator in conservatively treated distal radius fractures.

**Supplementary Information:**

The online version contains supplementary material available at 10.1186/s12891-026-09879-7.

## Introduction

Distal radius fractures are among the most frequently encountered injuries in adult orthopedic practice. Although surgical fixation has become increasingly common, conservative treatment remains an established option for fractures meeting acceptable stability and alignment criteria. Functional recovery following nonoperative management is generally favorable; however, patient-reported outcomes demonstrate substantial interindividual variability, even among fractures treated according to similar protocols [1, 2]. 

Patient-related factors are increasingly recognized as important determinants of outcome after fracture treatment [3]. Among these, obesity has attracted growing attention due to its association with altered bone metabolism, chronic low-grade inflammation, and reduced physical capacity [4]. Elevated body mass index (BMI) has been linked to inferior outcomes in several orthopedic conditions, including lower extremity fractures and joint arthroplasty [5, 6]. However, its role in upper extremity trauma, particularly in conservatively treated fractures, is less clear. In daily practice, obesity is often perceived as a potential risk factor for suboptimal recovery, yet the evidence supporting this assumption remains inconsistent.

In distal radius fractures, fracture pattern and patient age are well-established predictors of functional outcome. More complex fractures, as classified by the AO system, are generally associated with worse results, even when treated appropriately. Similarly, increasing age has been shown to correlate with higher disability scores and slower functional recovery [7, 8]. Whether BMI contributes additional prognostic information beyond these established factors is still uncertain. Many previous studies have either focused on surgically treated fractures or lacked adequate adjustment for confounding variables, making it difficult to isolate the independent effect of obesity [9–11]. 

The aim of the present study was to evaluate the relationship between BMI and functional outcomes in patients with distal radius fractures treated conservatively. Specifically, we sought to determine whether BMI independently influences 12-month DASH scores after controlling for age, sex, and fracture type. We hypothesized that while higher BMI may be associated with worse unadjusted functional outcomes, it would not remain an independent predictor when key clinical variables are taken into account.

## Materials and methods

### Study design and patient selection

This retrospective cohort study was conducted at a single tertiary trauma center between January 2023 and December 2025 following approval by the local institutional review board. During the study period, a total of 188 adult patients were treated for distal radius fractures at our institution. Of these, 141 patients (75%) were managed conservatively with closed reduction and immobilization, while 47 patients (25%) underwent surgical fixation based on fracture instability and standard treatment indications.

The present study focused exclusively on patients treated nonoperatively in order to evaluate functional outcomes within a homogeneous conservative treatment cohort. Inclusion criteria were adult patients with a distal radius fracture managed nonoperatively and availability of functional outcome data at 12 months of follow-up. Patients were excluded if they underwent surgical fixation at any stage during follow-up, had associated fractures of the same extremity, sustained pathological fractures, or had incomplete demographic or outcome data. Patients who experienced loss of reduction during follow-up and subsequently required surgical fixation were excluded from the study.

Among the 141 conservatively treated patients initially identified, 12 (8.5%) were excluded due to incomplete 12-month DASH data. Consequently, 129 patients with complete 12-month DASH scores were included in the final analysis. As this was a retrospective study, no a priori sample size calculation was performed. Instead, all eligible patients treated during the study period were included.

### Fracture classification and treatment protocol

All fractures were classified according to the AO/OTA classification system using initial radiographs. Due to the limited number of type C fractures, AO fracture types were grouped as type A versus type B/C for statistical analysis [12]. 

Nonoperative management was selected according to contemporary evidence-based criteria and accepted standards of care. Conservative treatment was considered appropriate in cases demonstrating acceptable alignment and stability, including dorsal tilt < 10–15°, radial height loss < 5 mm, intra-articular step-off < 2 mm, and absence of significant fracture instability following closed reduction. Additional considerations included patient age, functional demand, comorbidities, and ability to comply with follow-up.

Although AO type B fractures are often considered relatively unstable, selected cases demonstrating acceptable alignment and stability after reduction were managed nonoperatively according to established treatment principles.

Importantly, BMI was not used as a criterion in treatment decision-making, and treatment allocation was based solely on radiographic and clinical parameters rather than patient body habitus.

Conservative treatment consisted of closed reduction when indicated, followed by immobilization in a below-elbow cast in accordance with institutional protocols. Reduction technique, type of immobilization, and duration of casting were determined by the treating orthopedic surgeon based on fracture characteristics and stability. Radiographic evaluation was performed during follow-up to monitor maintenance of alignment and fracture healing.

Following cast removal, patients were instructed to initiate range-of-motion exercises. Formal physiotherapy was prescribed when clinically indicated at the discretion of the treating physician [13, 14]. 

### Follow-up

All patients were followed through routine in-person outpatient clinic visits at our institution, which functions as the primary referral center for the region. The 12-month DASH questionnaires were completed during these visits.

### Data collection

Demographic data including age and sex were recorded. Height and weight were obtained from medical records, and body mass index (BMI) was calculated as weight in kilograms divided by height in meters squared (kg/m²). BMI was analyzed both as a continuous variable and as a categorical variable according to World Health Organization criteria: normal weight (< 25 kg/m²), overweight (25–29.9 kg/m²), and obese (≥ 30 kg/m²) [15]. 

Functional outcome was assessed using the Disabilities of the Arm, Shoulder and Hand (DASH) questionnaire at 12 months after injury. The DASH score ranges from 0 to 100, with higher scores indicating greater disability [16, 17]. 

### Statistical analysis

Statistical analyses were performed using Python-based statistical software packages. The distribution of continuous variables was assessed using the Shapiro–Wilk test. Non-normally distributed continuous variables were summarized as median with interquartile range (IQR), while normally distributed variables were reported as mean ± standard deviation. Categorical variables were presented as frequencies and percentages.

Comparisons of DASH scores among BMI categories were performed using the Kruskal–Wallis test. When significant differences were detected, post hoc pairwise comparisons were conducted using the Mann–Whitney U test with Holm correction for multiple comparisons. The association between BMI and 12-month DASH score was evaluated using Spearman correlation analysis.

To assess the independent effect of BMI on functional outcome, a multivariable linear regression model was constructed with the 12-month DASH score as the dependent variable. Independent variables included BMI, age, sex, and AO fracture type (B/C vs. A). Robust standard errors (HC3) were used to account for potential heteroskedasticity. Statistical significance was set at *p* < 0.05. Missing data were handled using complete case analysis. Patients without available 12-month DASH scores were excluded, and no imputation methods were applied.

## Results

A total of 141 patients with conservatively treated distal radius fractures were initially identified. Of these, 129 patients with available 12-month DASH scores were included in the final analysis. The mean age of the study population was 50.3 ± 15.3 years, with a median age of 51 years (IQR 41–60). The mean BMI was 27.6 ± 4.5 kg/m² (median 27.06, IQR 24.49–30.67). The mean 12-month DASH score was 14.63 ± 10.95, with a median of 11.25 (IQR 6.75–22.50).

The distribution of AO fracture subtypes was as follows: A2 (70.5%), A3 (12.4%), B1 (7.0%), B2 (3.9%), B3 (3.1%), and C1 (3.1%) (Supplementary Table 1).

## Association between BMI and functional outcome

A weak but statistically significant positive correlation was observed between BMI and 12-month DASH score (Spearman ρ = 0.255, *p* = 0.0036), indicating higher disability scores with increasing BMI.

When analyzed categorically, DASH scores differed significantly among BMI groups (Kruskal–Wallis test, *p* = 0.0071). Post hoc analysis demonstrated that patients in the obese group had significantly higher DASH scores compared with those in the normal BMI group (Holm-adjusted *p* = 0.0054). The median 12-month DASH score was 9.00 (IQR 4.50–11.25) in the normal BMI group, 10.17 (IQR 6.75–25.00) in the overweight group, and 15.75 (IQR 10.71–27.25) in the obese group. No statistically significant differences were observed between the normal and overweight groups or between the overweight and obese groups.

### Patient characteristics according to BMI categories

Demographic and clinical characteristics stratified by BMI category are presented in Table 1. Age distribution did not differ significantly among BMI groups (*p* = 0.1067). Female sex was more prevalent in higher BMI categories (*p* = 0.0058). The distribution of AO fracture types (A vs. B/C) was similar across BMI groups (*p* = 0.8923).

**Table 1:** Demographic and clinical characteristics according to BMI Category

BMI Category	*n*	Age (years), mean ± SD	Female, *n* (%)	AO Type A, *n* (%)	DASH (12 months), median (IQR)	BMI (kg/m²), mean ± SD
Normal (< 25)	39	45.5 ± 17.1	15 (38.5%)	33 (84.6%)	9.00 (4.50–11.25)	22.7 ± 1.8
Overweight (25–29.9)	54	52.1 ± 14.7	28 (51.9%)	45 (83.3%)	10.17 (6.75–25.00)	27.4 ± 1.5
Obese (≥ 30)	36	52.7 ± 13.1	27 (75.0%)	29 (80.6%)	15.75 (10.71–27.25)	33.3 ± 2.8

Between-group comparisons revealed no significant differences in age (Kruskal–Wallis test, *p* = 0.1067) or AO fracture type distribution (A vs. B/C; chi-square test, *p* = 0.8923). The proportion of female patients differed significantly across BMI categories (chi-square test, *p* = 0.0058). DASH scores at 12 months showed a significant difference among BMI groups (Kruskal–Wallis test, *p* = 0.0071)

### Multivariable analysis

Results of the multivariable linear regression analysis for 12-month DASH score are shown in Table 2. After adjustment for age, sex, and AO fracture type, BMI was not independently associated with functional outcome (β = 0.283, 95% CI − 0.151 to 0.716; *p* = 0.201). Increasing age was independently associated with higher DASH scores (β = 0.307 per year, *p* < 0.0001). Patients with AO type B or C fractures demonstrated significantly worse functional outcomes compared with those with type A fractures (β = 6.777, *p* = 0.0081). Sex was not significantly associated with DASH score in the adjusted model.

**Table 2:** Multivariable linear regression analysis for 12-Month DASH score

Predictor	β Coefficient	95% Confidence Interval	*p*-value
Intercept	-9.610	-22.147 to 2.926	0.1330
BMI (kg/m²)	0.283	-0.151 to 0.716	0.2010
Age (years)	0.307	0.185 to 0.429	< 0.0001
Sex (Male = 1)	-0.305	-4.189 to 3.579	0.8777
AO Type (B/C vs. A)	6.777	1.759 to 11.794	0.0081

In the multivariable model including BMI, age, sex, and AO fracture type (B/C vs. A), BMI was not independently associated with the 12-month DASH score (β = 0.283, 95% CI − 0.151 to 0.716; *p* = 0.201). Increasing age (β = 0.307, *p* < 0.0001) and AO type B/C fractures (β = 6.777, *p* = 0.0081) were independently associated with higher (worse) DASH scores

## Discussion

The principal finding of this study is that although higher BMI was associated with worse unadjusted functional outcomes following conservative treatment of distal radius fractures, BMI did not remain an independent predictor after adjustment for age and fracture severity. In contrast, increasing age and AO type B/C fractures demonstrated independent associations with worse 12-month DASH scores.

Importantly, while the difference in DASH scores between normal-weight and obese patients reached statistical significance, the absolute magnitude of this difference (approximately 6–7 points) falls below the commonly reported minimal clinically important difference (MCID) for the DASH score, which is approximately 10–15 points. Therefore, the clinical relevance of this association should be interpreted cautiously [16, 17]. 

The association between obesity and inferior musculoskeletal outcomes has been well documented in the orthopedic literature. Proposed mechanisms include altered bone quality, impaired microvascular circulation, chronic low-grade inflammation, and reduced physical conditioning, all of which may negatively influence fracture healing and rehabilitation [4, 18–22]. In the context of distal radius fractures, however, the upper extremity is not directly load-bearing, and the biomechanical impact of increased body weight may therefore be less pronounced compared with lower extremity fractures [23]. This may partially explain why BMI demonstrated a significant association with DASH scores in univariate analyses but failed to retain independent significance in the multivariable model.

Age and fracture pattern were the strongest determinants of functional outcome in this conservatively treated cohort. Increasing age was independently associated with higher disability scores, consistent with prior reports demonstrating slower functional recovery in older patients. Similarly, AO type B/C fractures were linked to worse outcomes compared with type A fractures, reflecting greater fracture complexity.

The relatively younger mean age of our cohort likely reflects the demographic characteristics of our urban trauma population. While distal radius fractures are commonly associated with elderly osteoporotic individuals, regional variation exists; therefore, caution is warranted when extrapolating these findings to predominantly elderly populations.

From a clinical standpoint, our findings suggest that BMI alone should not be considered an independent determinant of long-term functional outcome in patients treated conservatively for distal radius fractures. Established factors such as fracture severity and patient age appear to play a more substantial role. These results do not imply that BMI is irrelevant, but rather that its effect may be mediated through other clinical variables.

Several limitations must be acknowledged. First, the retrospective design limits control over treatment variables and introduces potential selection bias. Second, although treatment decisions were based on standardized radiographic criteria, detailed radiographic measurements such as dorsal tilt, radial height, and intra-articular step-off were not systematically recorded and therefore could not be incorporated into the regression model, which may have introduced residual confounding. Third, the unequal distribution of sex across BMI categories, particularly the higher proportion of female patients in the obese group, may have introduced bias despite adjustment in the multivariable model. Fourth, only 12-month functional outcomes were available, preventing assessment of early recovery trajectories. Finally, although the cohort represents the full eligible population during the study period, the sample size may have been insufficient to detect small independent effects of BMI, raising the possibility of a type II error.

Despite these limitations, this study provides valuable insight into the role of BMI in conservatively treated distal radius fractures. Strengths include the use of a validated patient-reported outcome measure, inclusion of AO fracture classification, and application of multivariable analysis to account for key confounders. By focusing exclusively on nonoperatively treated patients, the study addresses a clinically relevant population that has been underrepresented in prior research.

## Conclusion

In conservatively treated distal radius fractures, higher BMI was associated with worse unadjusted functional outcomes but did not independently predict 12-month DASH scores after adjustment for age and fracture severity. These findings suggest that BMI alone may have limited value as an independent prognostic indicator in conservatively treated distal radius fractures.

## Supplementary Information

Supplementary Material 1.

## Data Availability

The datasets generated and/or analyzed during the current study are not publicly available but are available from the corresponding author on reasonable request.
