# Supplementary material for: Does body mass index independently affect functional outcomes after conservatively treated distal radius fractures? a retrospective cohort study
Source: BMC Musculoskelet Disord. 2026 Apr 22;27:480. doi: 10.1186/s12891-026-09879-7 (PMC13237960; doi:10.1186/s12891-026-09879-7)
Supplement: Supplementary file 1 — Supplementary Material 1. [file 12891_2026_9879_MOESM1_ESM.docx]

## Supplementary Table 1. Distribution of AO/OTA fracture subtypes in the study population

| AO subtype | n | % |
| --- | --- | --- |
| A2 | 91 | 70.5 |
| A3 | 16 | 12.4 |
| B1  B2  B3  C1  Total | 9  5  4  4  129 | 7  3.9  3.1  3.1  100 |
|  |  |  |

* Although AO type B3 and C1 fractures are typically considered unstable, selected cases with acceptable post-reduction alignment and stability were managed nonoperatively with close follow-up.
